# Vertical gastric plication versus Nissen fundoplication in the treatment of gastroesophageal reflux in children with cerebral palsy

**DOI:** 10.1590/S1516-31802007000100004

**Published:** 2007-01-04

**Authors:** Antonio Paulo Durante, Sergio Tomaz Schettini, Djalma José Fagundes

**Keywords:** Fundoplication, Gastroesophageal reflux, Cerebral palsy, Muscle spasticity, Child, Fundoplicatura, Refluxo gastroesofágico, Paralisia cerebral, Espasticidade muscular, Criança

## Abstract

**CONTEXT AND OBJECTIVE:**

Association between neurological lesions and gastroesophageal reflux disease (GERD) in children is very common. When surgical treatment is indicated, the consensus favors the fundoplication technique recommended by Nissen, despite its high morbidity and relapse rates. Vertical gastric plication is a procedure that may have advantages over Nissen fundoplication, since it is less aggressive and more adequately meets anatomical principles. The authors proposed to compare the results from the Nissen and vertical gastric plication techniques.

**DESIGN AND SETTING:**

Randomized prospective study within the Postgraduate Surgery and Experimentation Program of Unifesp-EPM, at Hospital do Servidor Público Estadual (IAMSPE) and Hospital Municipal Infantil Menino Jesus.

**METHODS:**

Fourteen consecutive children with cerebral palsy attended between November 2003 and July 2004 were randomized into two groups for surgical treatment of GERD: NF, Nissen fundoplication (n = 7); and VGP, vertical gastric plication (n = 7). These were clinically assessed by scoring for signs and symptoms, evaluation of esophageal pH measurements, duration of the operation, intra and postoperative complications, mortality and length of hospital stay.

**RESULTS:**

The mean follow-up was 5.2 months; symptoms were reduced by 42.8% (NF) (p = 0.001) and 57.1% (VGP) (p = 0.006). The Boix-Ochoa score was favorable for both groups: NF (p < 0.001) and VGP (p < 0.042). The overall mortality was 14.28% in both groups and was due to causes unrelated to the surgical treatment.

**CONCLUSION:**

The two operative procedures were shown to be efficient and efficacious for the treatment of GERD in neuropathic patients, over the study period.

## RESUMO

**CONTEXTO E OBJETIVO:**

A associação de crianças portadoras de lesão neurológica com doença do refluxo gastroesofágico (DRGE) é muito freqüente. Quando indicado o tratamento operatório, o consenso é pela técnica de fundoplicatura preconizada por Nissen, porém com alta taxa de morbidade e recidiva. A técnica de plicatura gástrica vertical é um procedimento que tem eventuais vantagens sobre a técnica consensual por ser menos agressiva e atender mais adequadamente aos princípios anatômicos. Os autores se propõem a comparar os resultados da técnica à Nissen com a de plicatura gástrica vertical.

**TIPO DE ESTUDO E LOCAL:**

Estudo prospectivo randomizado do Programa de Pós-graduação em Cirurgia e Experimentação da Universidade Federal de São Paulo (UNIFESP-EPM), Hospital do Servidor Público Estadual-Iamspe e Hospital Municipal Infantil Menino Jesus.

**MÉTODOS:**

14 crianças consecutivamente internadas com paralisia cerebral no período de novembro de 2003 a julho de 2004 foram randomizadas em dois grupos para o tratamento cirúrgico da DRGE: Grupo FN (n = 7), fundoplicatura a Nissen, e Grupo PGV (n = 7), plicatura gástrica vertical. Foram avaliados clinicamente através de um escore de sinais e sintomas, a avaliação da pHmetria esofágica, o tempo operatório, complicações intra e pós-operatórias, mortalidade e a permanência hospitalar.

**RESULTADOS:**

O acompanhamento médio foi de 5,2 meses; redução dos sintomas em 42,8% (G-FN) (p = 0,001) e 57,1% (G-PGV) (p = 0,006). Escore de Boix-Ochoa foi favorável nos dois grupos G-FN (p < 0,001) e G-PGV (p < 0,042). A mortalidade global foi de 14,28% nos dois grupos e deveu-se a causas estranhas ao tratamento cirúrgico.

**CONCLUSÃO:**

Os dois procedimentos operatórios mostraram-se efetivos no tratamento da DRGE em pacientes neuropatas, no período de tempo estudado.

## INTRODUCTION

Gastroesophageal reflux disease (GERD) in infancy presents significantly greater incidence in the population with associated neurological disease. There is a consensus that multifactorial events are involved in its etiopathogenesis,^1,2^ which makes it difficult to evaluate the results from treatment and to obtain adequate uniformity regarding the most appropriate therapeutic conduct for children with serious cerebral palsy and GERD.

The conventional clinical treatment is often ineffective. Surgical treatment, based on carrying out different procedures for introducing anti-reflux valves, is surrounded by high morbidity and mortality.^3,4^ Children with neurological lesions and GERD present high rates of postoperative complications such as dysphagia, delayed stomach emptying, gas retention syndrome and infections.^5-7^ The reoperation rate reported in the literature for these patients ranges from 20 to 47%,^4,8^ whereas for patients without neurological lesions these rates are between 4 and 11%.^9,10^

With regard to surgical therapy, a clear preference is shown in the literature for the Nissen fundoplication (NF) procedure (complete enveloping of the abdominal esophagus by the gastric fundus). However, complications are observed with this procedure, even when treating non-neuropathic children.^5^

Collis (1957)^11^ described a gastroplasty technique for lengthening the esophagus in patients with hiatal hernia and short esophagus. This concept gained wide acceptance and was much utilized at that time, before it was understood that progressive shortening of the esophagus secondary to esophagitis can occur. Modifications to this technique were proposed, such that vertical gastric plication (VGP) would be performed without sectioning the gastric wall, with the association of a valve mechanism.^12-15^ Performing the plication produces lengthening of the abdominal esophagus, and fundoplication would offer better control over reflux. The observations have shown that fundoplication makes it difficult for the stomach to distend, thereby causing additional difficulty in passage through the esophagus and accentuating the occurrence of dysphagia and respiratory difficulties.^16-18^

Taylor et al. (1989)^19^ proposed that VGP should be performed via an abdominal route, with any type of associated fundoplication. They argued that this was a rapidly performed, simple and safe technique that diminished the possibility of the postoperative complications that are so frequent with the other techniques.

The various types of gastric fundoplication, especially those that are constructed right round the esophagus, can be considered to be an additional obstacle to passage through the esophagus, because they impose an angle on the esophagogastric transition and cause difficulty in the peristalsis occurring in the final portion of the esophagus. This inconvenience becomes felt more strongly among individuals with esophageal dysmotility, especially those with neuropathy, in whom such dysmotility is more severe.^5,6^

Thus, control over gastroesophageal reflux disease (GERD) in patients with dysmotility should be carried out through an operative procedure that alters the anatomy and physiology of the esophagogastric transition as little as possible. Alterations of this nature are promoted by NF, given the wrapping of the distal esophagus by the gastric walls.

Such alterations and the consequent discomfort induced by them are well known in clinical practice. Thus, it is routine practice for the majority of surgeons treating adults with GERD by means of NF to recommend that non-neuropathic patients (i.e. those with normal esophageal motility) should follow a pasty or even liquefied diet for the first two or three months after the operation.

The same postoperative conduct is recommended by pediatric surgeons, after performing NF to treat GERD in non-neuropathic patients. It should also be remembered that it is not uncommon in pediatric surgical practice to observe esophageal obstruction due to food, which is almost always transitory but may sometimes require urgent endoscopic treatment, as a result of non-observance of these recommendations.

Therefore, the proposal for the present study was to prospectively investigate a technique that does not have the inconveniences indicated in relation to passage through the esophagus, among patients with cerebral palsy. Our opinion was that VGP might be a good choice: this produces lengthening of the abdominal esophagus and makes reflux difficult, without the factors unfavorable to passage through the esophagus and lack of success from treatment that have been attributed to concomitant plication.

The VGP procedure would be compared with the technique proposed by Nissen. The latter technique is reported to be the one most frequently utilized among such patients.^20^

It should be added that no randomized prospective studies on the surgical treatment of GERD in neuropathic children were located in a review of the literature.^20^

## OBJECTIVE

This study was performed to compare the effectiveness of surgical treatment for gastroesophageal reflux by means of vertical gastric plication and by means of Nissen fundoplication, in children with cerebral palsy.

## METHODS

This randomized prospective study was conducted in two hospitals in the city of São Paulo: Hospital Municipal Infantil Menino Jesus and Hospital do Servidor Público Estadual Francisco Morato de Oliveira. The research was assessed and approved by the research ethics committees of the respective hospitals.

The patients were divided into two groups, the NF group (n = 7) and the VGP group (n = 7).

The inclusion criteria were that the subjects should be children within the age range of 0 to 17 years, with non-progressive chronic encephalopathy presenting predominance of motor abnormalities (cerebral palsy), and with GERD documented by means of prolonged measurement of esophageal pH. It was also necessary for the persons legally responsible for the children to have granted their free and informed consent to the procedures.

The exclusion criteria were that the subjects should not have chronic non-progressive encephalopathy presenting predominance of abnormalities of intelligence or behavior.

For all patients, a barium contrast examination was performed on the esophagus, stomach and duodenum, using the methodology of McCauley,^21^ to rule out possible anatomical causes of the GERD.

The two groups were compared by means of clinical criteria, measurement of esophageal pH, radiological examination, presence and evaluation of intra and postoperative complications, duration of the surgical operation and length of hospital stay.

The patients included in the study underwent clinical assessment by means of qualitative staging based on the 15 parameters that are most commonly associated with GERD in children (vomiting, regurgitation, nausea, irritability, dysphagia, pain, recurrent otitis, recurrent sinusitis, recurrent bronchopneumonia, apnea, wheezing in the chest, night coughing, stridor, anemia and hematemesis), before the operation and 30, 90 and 180 days after the operation, from information supplied by the caregivers responsible for the children. Scores were compiled according to the presence or absence of each of the 15 parameters included, and noting whether each parameter had the same value over the preceding 30 days.

Prolonged measurement of esophageal pH was carried out before the operation and 30, 90 and 180 days after the operation. The Scophe apparatus, version 1.4 (Dynamed^®^) with antimony electrode (Mediplus catheter), was utilized according to the methodology proposed by Boix-Ochoa et al.^22^ In this, the evaluation consists of counting the scores for five different parameters: percentage (%) of the time for which the pH remained less than 4.0 over the whole monitoring period; number of reflux episodes observed over a 24-hour period; number of reflux episodes lasting for more than five minutes; and duration of the most prolonged reflux episode. Events were considered to be gastroesophageal reflux when the measured pH was less than 4.0 for more than 15 seconds.

The medication utilized for preoperative sedation was oral administration of a midazolam solution (0.5 mg/kg), applied 30 minutes before the surgical procedure. The induction of anesthesia was carried out by means of inhalation of sevoflurane (Sevorane^®^) (6.0%) or halothane (2.0%), followed by administering of opioid (fentanyl 2.0 mcg/kg) and muscle relaxant (cisatracurium 0.5 mg/kg) for orotracheal intubation and placing the patient on controlled mechanical ventilation.

One dose of first-generation cephalosporin (50 mg/kg) was applied in the surgical theater prior to the surgical incision. Whether this medication was continued depended on the patient's clinical condition.

The type of surgical procedure was randomized immediately before the intervention in the surgical theater, by means of tossing a coin.

All the procedures were performed by the same surgeon and team.

Access to the abdominal cavity was obtained by medial xiphoumbilical incision, with the patient in horizontal dorsal decubitus. The abdominal part of the esophagus was identified and freed after opening up the phrenoesophageal membrane and partially sectioning of the hepatogastric ligament. The greater curvature of the stomach was mobilized by means of sectioning the gastrosplenic ligament and ligating the first short vessels. The arms of the diaphragmatic pillar were brought together using separate stitches of non-absorbable thread when they were patent for more than three centimeters.

**Figure 1:**
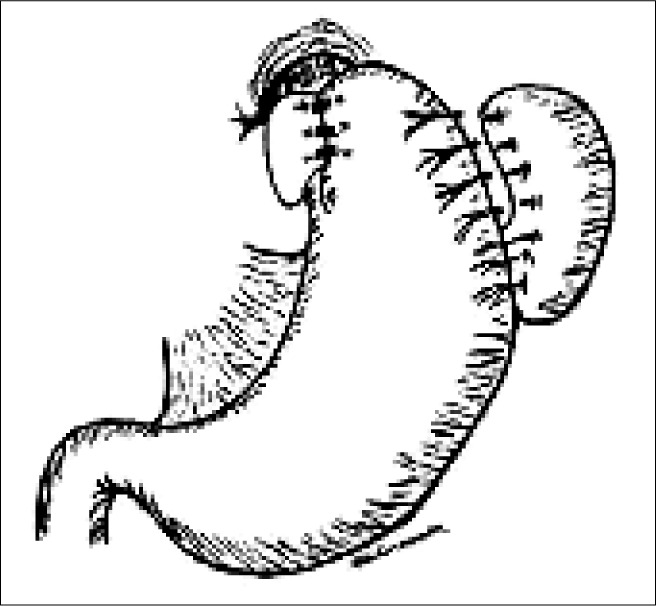
Diagram of the Nissen fundoplication procedure.

For the patients in the NF group, the abdominal segment of the esophagus was enveloped posteriorly by the gastric fundus, positioned in such a way as to wrap the whole circumference of the abdominal segment of the esophagus. This plication was attached using three separate stitches of non-absorbable thread: two cranial stitches, joining together stomach-esophagus-stomach; and one caudal stitch, joining stomach-stomach. The plication was kept close to the diaphragmatic pillar using one stitch of non-absorbable thread (Figure 1). A probe of caliber 16 to 18 Fr was kept between the esophagus and the stomach while the stitches were being applied, so as to ensure that the procedure was loose and did not constrict the esophagus in the abdomen.

**Figure 2:**
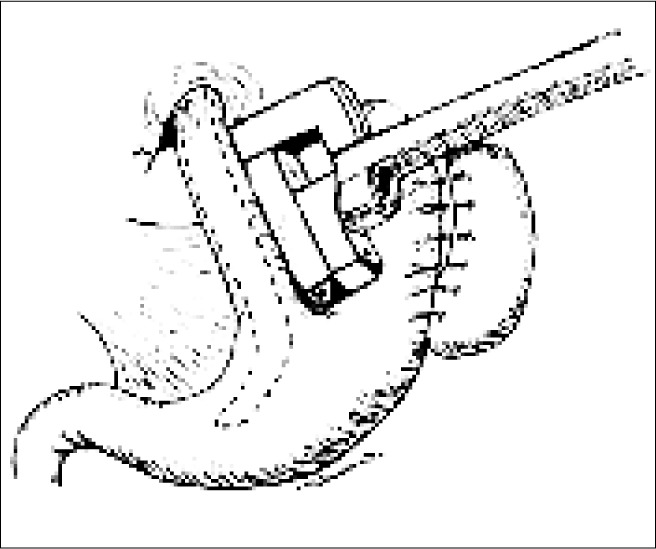
Diagram of the initial positioning of the linear stapler.

**Figure 3:**
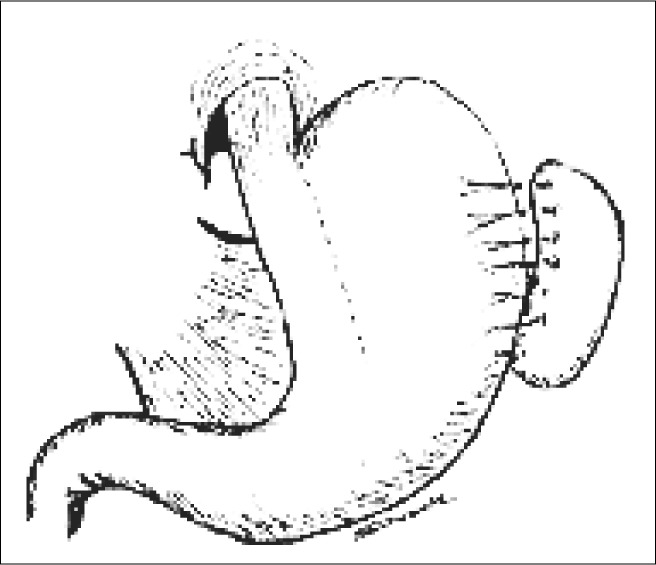
Diagram of the end of the vertical gastric plication procedure.

For the patients in the VGP group, a gastric lavage tube was inserted, of caliber similar to that of the esophagus. This was followed by stapling, using the TL-60 linear stapler (Ethi-con Endo-surgery^®^ Inc, Somerville, NJ, USA), starting at the lateral margin of the esophagogastric transition and extending parallel to the lesser gastric curvature or six centimeters. These titanium staples went through the full thickness of the stomach, thereby creating a division in the stomach without sectioning it (Figure 2). In this extension to the esophagus, a ratio of 4:1 was maintained between the neoesophageal length and width (Figure 3).

At the end of the procedure, the gastric lavage tube that had been shaping the esophagus was removed and was substituted by a nasogastric probe appropriate for the patient's size.

Because of clinical particularities, two children in the NF group (28.57%) underwent gastrostomy and two (28.57%) underwent gastrostomy and tracheostomy. Four children in the VGP group (57.14%) underwent gastrostomy.

All the patients underwent postoperative radiological evaluation.

A standardized record card was utilized for recording the duration of the surgical operation (from the time of the incision to the time of putting in the last stitch in the skin), the length of hospital stay and any intra and postoperative complications.

To perform the statistical calculations, the Statistical Package for Social Sciences (SPSS) for Windows version 10.0 software was utilized. Results with descriptive levels of less than 0.05 were considered to be statistically significant.

For analysis of the qualitative variables, the Pearson chi-squared and Fisher exact tests were utilized. Quantitative variables were analyzed by means of the Kolmogorov-Smirnov normality, Student t and Mann-Whitney tests. The variance of repeated measurements was analyzed by means of Anova and the Bonferroni multiple comparison method.

## RESULTS

### General features

From November 2003 to July 2004, 14 neuropathic children received surgical treatment for controlling GERD. Among these children there were nine boys (64.28%) and five girls (35.72%); their mean age was 67.78 months (range: 4 to 147 months). The average length of follow-up was 5.2 months.

### Symptomatic and etiopathogenic diagnoses

Table 1 shows the patient distribution in the NF and VGP groups, according to the symptomatic and etiopathogenic diagnoses, following the classification by Rosemberg.^23^

**Table 1.** Patient distribution in the Nissan fundoplication (NF) and vertical gastric pla- cation (VGP) groups, according to the symptomatic and etiopathogenic diagnoses, following the classification by Rosemberg^23^

	NF group	VGP group
**Symptomatic diagnosis**		
Diplegic spastic cerebral palsy	2	2
Tetraplegic spastic cerebral palsy	5	5
**Etiopathogenic diagnosis**		
Hypoxic-ischemic disease	6	6
Infectious disease	0	1
Central nervous system malformation	1	0

### Associated affections

Table 2 shows the patient distribution in the NF and VGP groups, according to associated affections.

**Table 2.** Patient distribution in the Nissan fundoplication (NF) and vertical gastric placation (VGP) groups, according to associated affections

Associated affections	NF group	VGP group
Laryngotracheomalacia	2	1
Hydrocephalus	2	3
Microcephaly	1	2
Macrocephaly	1	0
Periventricular leukomalacia	0	1
West's syndrome	0	2
Congenital luxation of the hip	1	0
Congenital talipes	1	0
*Pectus Carinatum*	1	0
Congenital cardiovascular malformation	0	3
Down's syndrome	0	1
Congenital glaucoma	0	1
Pulmonary hypertension	0	1

### Clinical score

**Figure 4:**
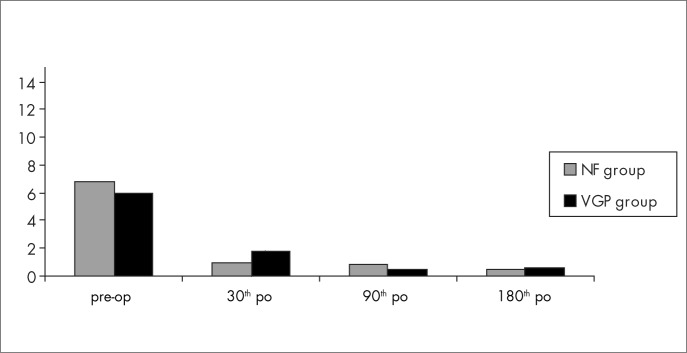
Patient distribution with regard to means of clinical scores for the Nissen fundoplication (NF) and vertical gastric plication (VGP) groups, measured before the operation and 30, 90 and 180 days after the operation (p < 0.05). Pre-op = preoperative; po = postoperative day.

There was a very significant reduction in the clinical scores for both the NF group (p = 0.001) and the VGP group (p = 0.006), comparing the preoperative scores with the scores for the 30th, 90th and 180th days after the operation (Figure 4). All the patients had a clinical improvement, and 42.8% of the NF group and 57.1% of the VGP group were asymptomatic throughout the postoperative period studied.

### Evaltion of pH measurements

The pre and postoperative pH measurements showed that for the NF group the only variable not showing a significant difference was gastroesophageal reflux episodes lasting for more than five minutes. For the VGP group, there was no significant difference for the variables of total number of reflux episodes, total number of reflux episodes lasting for more than five minutes and longer reflux episodes. These results are shown in Table 3.

**Table 3.** Means and standard deviations for the prolonged pre and postoperative esophageal pH measurements (Boix-Ochoa et al.^22^ parameters), for the patients in the Nissan fundoplication (NF) and vertical gastric plication (VGP) groups

Measurements	NF group			VGP group		
	pre-op	post-op		pre-op	post-op	
Boix-Ochoa	53.93 ± 17.68	14.56 ± 23.80	*p < 0.001	72.83 ± 26.99	36.38 ± 19.61	*p = 0.041
% pH < 4	14.81 ± 5.69	4.33 ± 8.94	*p = 0.002	25.73 ± 10.25	12.07 ± 7.38	*p = 0.042
total number of gastroesophageal reflux episodes	11.28 ± 7.75	2.29 ± 3.46	*p = 0.012	6.35 ± 4.36	4.04 ± 1.88	p = 0.273
number of gastroesophageal reflux episodes > 5 min	7.38 ± 2.38	2.89 ± 5.36	p = 0.083	9.83 ± 4.06	4.84 ± 3.92	p = 0.081
longer gastroesophageal reflux episodes	10.35 ± 11.25	2.02 ± 2.72	*p = 0.044	16.17 ± 9.57	8.22 ± 6.25	p = 0.217

* *significant differences.*

### Radiological evaltion

Contrast radiological examination of the esophagus, stomach and duodenum showed the presence of a hiatal hernia in one patient in the NF group (14.28%). No obstructive causes were observed that could have been responsible for the gastroesophageal reflux. Aspiration of the contrast into the tracheobronchial tree occurred in two patients in the VGP group (28.56%).

### Intraopertive complicions

No patients in either group presented intraoperative complications.

### Postopertive complicions

Valve migration occurred in one patient in the NF group (14.28%) and there was a para-esophageal hernia in one patient in the VGP group (14.28%). These were considered to be major postoperative complications.

Minor postoperative complications only occurred in the VGP group (28.57%): one patient presented infection of the urinary tract during the immediate postoperative period, and two patients presented a episode of pneumonia after the 90^th^ postoperative day.

Two deaths (28.56%) occurred, one in each group (14.28%), which were unrelated to the surgical treatment. One child had congenital cardiac malformation, with pulmonary hypertension, and died as a result of complications from these affections. The other had Down's syndrome and presented progressive fungal septicemia and multiple organ and system failure.

### Durtion of the surgical procedure

There was no statistically significant difference between the NF and VGP groups with regard to the duration of the surgical procedure: 109.29 ± 26.52 minutes and 99.29 ± 19.67 minutes, respectively (p = 0.440).

### Length of hospital stay

There was no statistically significant difference between the NF and VGP groups with regard to the length of hospital stay: 7.33 ± 1.03 days and 7.67 ± 3.79 days, respectively (p = 0.894).

## DISCUSSION

The greater incidence of gastroesophageal reflux among children with neuropathy is due to various predisposing factors: increased intraabdominal pressure caused by the spasticity; anatomical deformity of the esophagogastric junction associated with thoracoabdominal kyphoscoliosis; reduced pressure in the lower esophageal sphincter; delayed stomach emptying; chronic pulmonary disease; hypotonia of the diaphragmatic pillar, and also esophageal, gastric and intestinal dysmotility inherent to the neurological disease.^1,24^

The clinical assessment of a child with neurological lesions depends on the ability of the persons legally responsible for the child to discern them, and on very specific criteria. It seemed to us to be more appropriate for the present study to opt for the utilization of a qualitative clinical score. When this scoring system was applied to the groups, both of them presented significant postoperative reduction in the parameters, in comparison with the preoperative values. We observed absence of symptomatology in more than half of the patients in the VGP group.

In the radiological examination, we were unable to detect any difference between the two surgical procedures.

The prolonged esophageal pH evaluations were a direct and objective method for measuring the exposure of the esophagus to the reflux of gastric juices. When this examination is performed over a prolonged period, it presents high sensitivity and specificity indices.^25^ The Boix-Ochoa methodology is considered to be the most appropriate for application to the pediatric age group.^22^

The patients’ indications for surgery were due to an association of symptoms related to gastroesophageal reflux and confirmation of such reflux outside of normal patterns, by means of prolonged esophageal pH measurement. Maintenance of clinical treatment for such patients with severe mental retardation for an indefinite time becomes impracticable. In addition to the predisposing factors associated with such lesions, when these children remain in dorsal decubitus the whole time, they are more liable to develop complications from gastroesophageal reflux.^26,27^

Surgical treatment for controlling gastroesophageal reflux has the objectives of lengthening the intra-abdominal portion of the esophagus, accentuating the acute esophagogastric angle and performing fundoplication to transmit the pressure from the gastric fundus to the zone of the lower esophageal sphincter.^28^

Complete gastric fundoplication, as in the NF procedure, is often complex from a technical point of view, because of the presence of thoracoabdominal deformities.

Performing NF in such patients may also result in complications like dysphagia, because of the difficulty placed on free passage through the esophagus due to the presence of both gastric walls posteriorly to the abdominal portion of the esophagus, thereby imposing an angle on it. This is in addition to wrapping the stomach around the esophagus, right round its circumference, thereby causing extra difficulty for peristalsis of the esophagus, which is already compromised by these patients’ dysmotility. This combination of factors may favor greater occurrence of pulmonary complications, which is confirmed by the high frequency with which it becomes necessary to perform tracheostomy in association with the plication. For many surgeons, this is performed routinely during the same surgical procedure.

The occurrence of gas retention syndrome following NF is found in up to 50% of cases.^20,24^ Esophageal dysmotility, in association with the construction of a valve around the intra-abdominal portion of the esophagus, is the main agent responsible for the greater complication rate in these patients.^29^ These symptoms may be persistent and difficult to treat, thus stimulating surgeons to seek new surgical alternatives.

**Figure 5:**
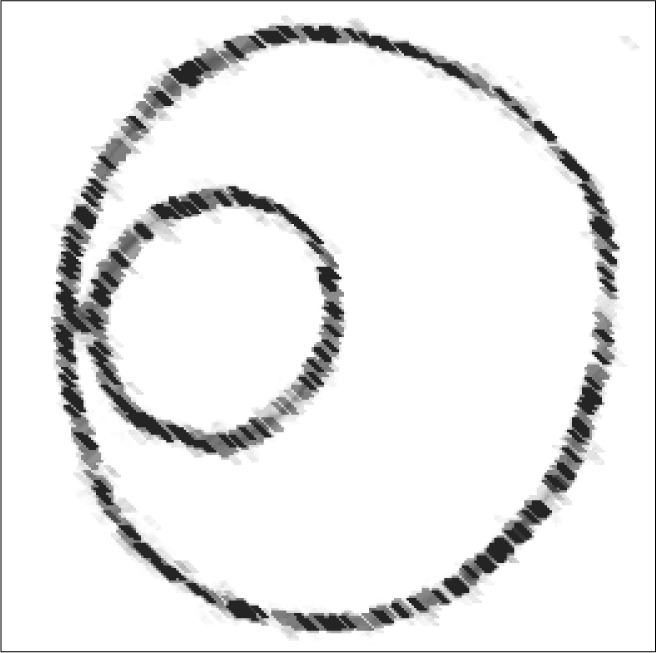
Schematic representation of vertical gastric plication (axial view).

**Figure 6:**
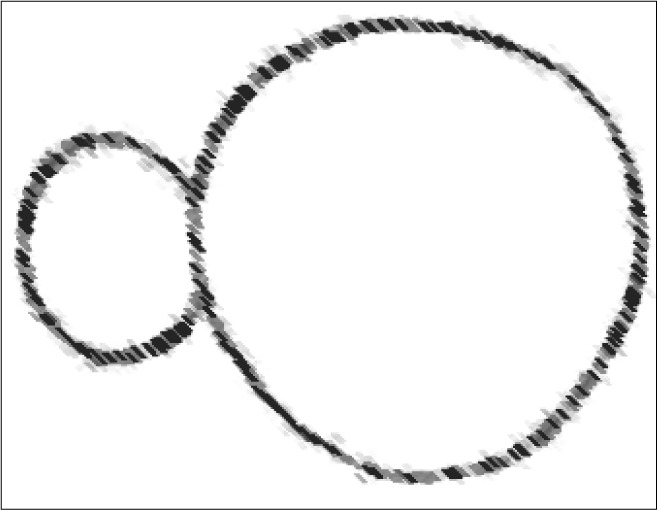
Schematic representation of Nissen fundoplication (axial view).

The alternative of VGP has the anatomophysiological basis of controlling gastroesophageal reflux by means of lengthening the intraabdominal portion of the esophagus, practically without causing alterations to esophagogastric anatomy. This occurs through making the entry angle into the gastric reservoir more acute, increasing the area of pliant wall capable of functioning as a valve and not interfering with the free emptying of the esophagus, because no type of valve is installed in association with this procedure. VGP follows the same physical principles as does NF, governed by Laplace's law (Figures 5 and 6).^10,27^

Laplace's law establishes that, in two hollow visceral tubes with walls of equal tension (T), the pressure (P) is greater in the visceral tube of smaller radius (R), since P = T/R. From this concept, it is easy to imagine that a narrowed tube created from the esophagogastric junction could function as a high-pressure zone, thereby impeding gastroesophageal reflux.^10,27^

Since the two surgical procedures follow the same physical principles, it can be believed that their control over the reflux would also be similar. In the present study, both surgical procedures were shown to be effective in controlling gastroesophageal reflux. There was improvement in all the parameters evaluated: in the NF group, only one parameter did not show statistical significance, while in the VGP group there was no statistical significance for three parameters.

The morbidity and mortality resulting from anti-reflux surgery on children with severe neurological lesions are considered to be high in comparison with results among children without such lesions.^29^ In the present study, the observations of a major postoperative complication rate of 14.28% in each group and a minor complication rate of 28.57% in the VGP group are at the lower limit of the rates reported in the literature. The two deaths that occurred in the present study were unrelated to the surgical procedure.

Some authors have observed that a greater rate of complications was expected to occur six months after the operation.^20,30^ It is possible that a greater difference in the present study might have been observed if there had been longer follow-up of these patients, since in this preliminary study the longest follow-up was for eight months after the operation.

Gastrostomy should be performed in children with severe deglutition disorders.^31,32^ In the present study, gastrostomy had to be performed in association with the anti-reflux procedure in 57.0% of the patients in the two groups. For another two patients in the NF group, tracheostomy was performed in association with the anti-reflux procedure because of these children's pulmonary conditions.

The two surgical procedures are similar, as are the degree of anesthetic and surgical aggression. This is shown in the duration of the operation and length of hospital stay, which did not differ between the two procedures, although it has been reported in the literature that VGP is a faster procedure involving shorter hospital stay.^10,19^ However, these two procedures have never been prospectively compared for treating gastroesophageal reflux in neuropathic children.^20^

Today, the access most recommended for surgical treatment of gastroesophageal reflux is by means of laparoscopy.^33,34^ We chose to perform these procedures via the conventional route, because of the difficulty in positioning the linear stapler to perform the vertical gastric plication. In the future, with more adequate materials and greater experience, it will be possible to perform these procedures via the laparoscopic route.

## CONCLUSION

Both surgical procedures were shown to be effective for controlling gastroesophageal reflux in patients with neurological lesions.

A greater number of cases and a longer follow-up period are needed in order to come to a conclusion as to which of the two procedures is better.
